# The presence of microorganisms in follicular fluid and its effect on the outcome of in vitro fertilization-embryo transfer (IVF-ET) treatment cycles

**DOI:** 10.1371/journal.pone.0246644

**Published:** 2021-02-08

**Authors:** Safiyya Faruk Usman, Ibraheem Rais Shuaibu, Korede Durojaiye, Nubwa Medugu, Kenneth Chukwuemeka Iregbu

**Affiliations:** 1 Department of Obstetrics and Gynaecology, National Hospital Abuja, Abuja, Nigeria; 2 Department of Medical Microbiology, National Hospital Abuja, Abuja, Nigeria; University of Mississippi Medical Center, UNITED STATES

## Abstract

**Objectives:**

To determine if the presence of microorganisms in follicular fluid of women undergoing in vitro fertilization-embryo transfer (IVF-ET) adversely affects the outcome of the treatment cycles.

**Methods:**

Follicular fluid was collected from 86 women enrolled for IVF-ET at the National Hospital, Abuja from June 1, 2018 to December 1, 2018. Microscopy imaging and cultures were performed to identify bacteria and fungi in the follicular fluid and the vagina. Women with follicular fluid microorganisms were the test group while the controls were those without follicular fluid microorganisms. Fertilization and pregnancy rates were subsequently determined and their association with the presence of follicular fluid microorganisms was assessed using univariable and multivariable logistic regression modelling.

**Results:**

The mean age and mean Body Mass Index (BMI) of the participants were 35(± 3.5) years and 28(± 4.9) kg/m^2^ respectively. Bacteria and fungi were isolated in 17% of the follicular fluid samples collected (n = 15/86). Most common isolates were Streptococcus spp. (n = 4/15), *Staphylococcus aureus* (n = 7/15), Enterococcus spp. (n = 4/15), Lactobacillus species (n = 2/15) and *Candida albicans* (n = 2/15). There was no statistically significant difference in the fertilization rates (adjusted odds ratio [AdjOR] 0.55, 95% confidence interval [CI] 0.04–7.34; P = 0.10) and pregnancy rates (AdjOR 4.02, 95% CI 0.56–28.92; P = 0.19) between the group of women with positive follicular fluid bacterial /fungal colonization as compared against those with negative follicular fluid colonization.

**Conclusion:**

Isolation of microorganisms from follicular fluid did not adversely affect fertilization and pregnancy rates following IVF-ET treatment cycles at the fertility centre of National Hospital, Abuja.

## Introduction

Until recently, the site of conception and fetal growth (upper genital tract) was believed to be sterile and that the baby was first exposed to microorganisms only during birth [1]. A number of studies have confirmed that many potentially pathogenic microorganisms frequently and transiently colonize the female upper genital tract in the absence of inflammatory disorders or symptomatic infection [2, 3].

The human follicular fluid (FF) which is the microenvironment of the oocyte during its development and maturation, is a semi-viscous, hypocoagulable fluid comprising a wide variety of biologically active molecules [4]. It is a product of transfer of plasma constituents across the blood-follicular barrier and the secretory activity of granulosa and theca cells [5]. Follicular fluid is important in ovarian physiology, including steroidogenesis, growth of the follicle and ovulation, maturation of the oocyte and its transport to the oviduct [6].

Multiple microorganisms have been isolated from follicular fluids collected at the time of trans-vaginal oocyte retrieval and these organisms have been classified as either contaminants or colonizers [2–4]. Contaminants have been defined as microorganisms present in both the FF (upper genital tract) and the vaginal (lower genital tract) samples collected of the same woman; while colonizers are microorganisms detected within the follicular fluid, but not within the vaginal sample [2, 7]. Microorganisms that are commonly found to be colonizing the follicular fluid were normal flora of the vagina (*Lactobacillus* spp.), gastrointestinal tract (*Bifidobacterium* spp., enteric bacteria, *S*. *agalactiae*), skin (*Staphylococcus* spp.) and oral mucosa (*Streptococcus* spp.) [8].

It has been shown that ovarian follicles can be colonized, and that the presence of microorganisms in follicular fluid collected at the time of trans-vaginal oocyte retrieval for in-vitro fertilization (IVF) can result in poor outcomes such as decrease in the number of oocytes retrieved, poor embryo quality, decrease in embryo transfer rates, pregnancy rates and live birth rates [4, 8].

As Assisted Reproductive Technology (ART) is still relatively new in Nigeria [9, 10], there is paucity of data on the effect of follicular fluid microorganisms on IVF outcome. There are studies from western-industrialized countries documenting adverse IVF outcomes when microorganisms are detected in follicular fluid but few in developing countries like Nigeria. This study aims to contribute to this growing area of research by exploring the adverse effects of follicular fluid microorganisms on outcome of IVF-ET treatment cycles. Measures to optimize treatment outcomes such as routine screening of follicular fluid and possibly antimicrobial prophylaxis may be advocated if an adverse outcome is found. It will also provide grounds for further research on routine screening of follicular fluid prior to fertility treatments and the relationship between follicular fluid microorganisms and unexplained infertility [8].

## Materials and methods

### Study setting and design

A prospective cohort study was conducted from June 1, 2018 to December 31, 2018, among 90 women scheduled for IVF-ET at the IVF centre, National Hospital Abuja (NHA). Consecutive consenting women aged 18 to 38 years with normal menstrual cycle (24–35 days) and normal uterine cavity confirmed by previous hysteroscopy or hysterosalpingography were enrolled. Women who had clinical evidence of cervicitis or endometritis, antibiotic therapy prior to commencement of ovarian hyperstimulation, factors that might affect reproductive outcome, such as previous history of ovarian surgery; endometriosis; endocrinological disorders (abnormal testosterone, abnormal prolactin, diabetes mellitus); significant cardiovascular, pulmonary, neurological, allergic, hepatic or renal disease were excluded from the study. Interviewer-administered questionnaire (S1 Text) was used to obtain the socio-demographic, gynaecological, obstetric and past medical history of the participants. Further information was collected from the medical records of the participants.

### Sample size

The minimum sample size (n) of 90 was determined by applying the formula for comparison of two proportions [11, 12] as illustrated in S2 Text. Eighty-six participants completed the study while 4 had cycle cancellation on account of poor response.

### Specimen collection

Specimens collected were high vaginal swabs (HVS) and samples of follicular fluid collected during trans-vaginal oocyte retrieval. The IVF treatments were carried out using the standard protocol as described by Elder and Dale [13] and Bhandari et al. [14] Pituitary down-regulation was achieved with a Gonadotrophin releasing hormone (GnRH) agonist injection while controlled ovarian hyper stimulation was achieved with variable amounts of human menopausal gonadotrophin (HMG), (between 75-300IU) or recombinant FSH 150IU daily (Bharat Serums and Vaccines Ltd, Ambarnath, India). Treatment was monitored by serial transvaginal ultrasound scans. Induction of ovulation was achieved with 5000–10,000IU of hCG (Bharat Serums and Vaccines Ltd, Ambarnath, India), when at least two to three follicles have attained a diameter of between 18-22mm [13].

Oocytes were retrieved 34–36 hours after hCG administration. It was an aseptic procedure carried out in the IVF theatre following the administration of local anaesthesia. Each participant was placed in lithotomy position and the vulva cleaned with normal saline. A sterile Cusco’s speculum was inserted into the vagina to expose the cervix and the vagina was also cleaned with gauze moistened with sterile normal saline. HVS were collected by pressing a sterile swab to the vaginal fornix and rotating four times, allowing contact for at least 30 seconds. A second swab was collected in the same manner. Further contact between the swab stick and the vaginal wall was avoided. Swabs were placed back into the swab container, and transported in a zip lock bag to the medical microbiology laboratory for processing within 15 minutes of collection.

Under trans-vaginal ultrasound guidance, the mature follicle(s) in both left and right ovaries were located and with the aid of a vacuum disposable needle attached to the ultrasound probe, the follicular fluid was aspirated into sterile tubes. The embryologists poured the content of the test-tube into the scanning dish, forming a thin layer of fluid which was viewed under a Leica® inverted microscope (objectives 5x-63x) for the presence of oocytes. This was carried out under a laminar hood with uniform temperature base, and sterile air flow.

The identified oocytes were transferred onto specially developed flushing media (Origio, Måløv, Denmark) at controlled environmental conditions while sample of the follicular fluid was immediately collected in a sterile universal bottle. It was placed in a zip lock bag and immediately transported to the medical microbiology laboratory for processing within 15 minutes of collection to avoid loss of the potential microorganisms present.

The oocyte morphology as well as its maturity were assessed by noting the volume, density and condition of the surrounding coronal and cumulus cells. If the oocyte was seen, the presence of a single polar body indicates that it has reached the stage of metaphase II. The oocytes were washed in pre-warmed (37°C) flushing medium, blood clots and granulosa cells were removed carefully by washing with a Pasteur pipette. The oocytes were immediately transferred to pre-equilibrated SAGE 1 culture medium (Origio, Måløv, Denmark) and stored at 37°C in a 5% CO_2_ incubator. They were allowed to rest for two hours and then inseminated with prepared semen maintaining stable temperature of 37°C and P^H^ of 7.2–7.3 when conventional IVF was used or denuded for ICSI in case of poor sperm quality.

The treated oocytes were returned back to the incubator for culturing and they were checked for evidence of fertilization on the following day, which was indicated by the presence of 2 pronuclei. The embryos were transferred to a fresh culture medium (SAGE 1) and returned to the incubator for further culture.

Embryo transfers were done on day 2 to 5 of the embryo culture using a Wallace Sure-Pro Ultra catheter® (Origio, Måløv, Denmark). All participants underwent fresh embryo transfers and the number of embryos transferred (2, 3 or 4) depended on the age of the woman and embryo quality. Luteal phase support was achieved with intravaginal progesterone pessary, Cyclogest® 400mg (Teva UK Ltd, Essex, England) per vaginum twice daily and Oestradiol Valerate 2mg (Progynova®; Bayer Plc, Berkshire, UK) orally twice daily. The cycle was cancelled if day 9–10 folliculometry revealed one or no developing follicle, if no oocytes were retrieved, or if fertilization failed.

### Isolation and identification of microorganisms from high vaginal swabs and follicular fluid

In the laboratory, a direct smear was made from one HVS on a glass slide and gram-stained to observe for the presence of bacteria and yeast cells. Follicular fluid was also gram-stained using standard methods [15]. Microorganisms are seen on a smear when the specimen contains at least 10^5^ organisms per millilitre [16]. The morphological characteristics (gram reaction, shape of cells and organization of cells in clusters, strings etc.) were noted which aided in identification of the microorganisms. The second HVS swab and some follicular fluid were inoculated separately unto Blood, Chocolate, MacConkey and Saboraud’s agar media. These were incubated overnight, with blood agar and chocolate agar in a candle extinction jar (3% to 5% CO2 achieved with this method) and MacConkey agar and Saboraud’s agar media in ambient air. Media plates with bacteria growth (positive plates) were sub-cultured unto MRS Lactobacillus agar, Yeast extract agar and Chromagar Orientation for identification of lactobacilli, yeasts and gram-negative enteric organisms. Further physio-biochemical tests were carried out to identify the isolates. These included motility testing, catalase test, indole test, urease test, germ tube test, oxidase test and an array of sugar fermentation tests. The result of the HVS culture and follicular fluid culture were retrieved within 48–72 hours for use in patient management where appropriate. Microorganisms isolated from follicular fluid were classified as: (1) ‘colonizers’ if microorganisms were detected within the follicular fluid, but not within the vaginal swab; or (2) ‘contaminants’ if microorganisms detected in the vaginal swab were also detected within the follicular fluid [8, 17].

A serum pregnancy test was done on the 14^th^ day post embryo-transfer and a positive pregnancy test is interpreted as pregnancy. Clinical pregnancy was diagnosed by ultra-sonographic visualization of one or more gestational sacs two weeks after serum pregnancy test [18]. There was no case of ectopic pregnancy. For the purpose of this study, follow-up ended with a negative pregnancy test or the detection of clinical pregnancy after a positive pregnancy test.

The primary outcomes were isolation of microorganisms from follicular fluid and the vagina. Secondary outcomes were; number of oocytes retrieved, number of embryos generated, fertilization rate (the number of fertilized eggs relative to the number of retrieved oocytes) [19], biochemical pregnancy rate (a pregnancy diagnosed only by the detection of β-hCG in serum or urine) [18], clinical pregnancy rate (the number of clinical pregnancies per 100 initiated cycles) [18].

### Statistical analysis

The information obtained from participants and the outcome were transferred from an excel spreadsheet to Stata 15.0 (Stata Corporation, College Station, Texas) statistical software for analyses. Frequency distributions of variables were generated and presented in tables and charts. Categorical variables such as fertilization and pregnancy rates were expressed as absolute numbers and percentages. Continuous variables such as age and BMI were described using mean and standard deviation (SD) while duration of infertility was described using median and interquartile range (IQR) and these variables were subsequently categorised. Chi-square test (or Fishers Exact test) were used to assess the relationship between the socio-demographic and gynecological characteristics and follicular fluid colonization.

For the logistic regression modelling, >50% was considered high fertilization rate while ≤50% was considered low fertilization rate [20]. Univariable and multivariable logistic regression modelling was conducted to evaluate the relationship between follicular fluid colonization and achieving fertilization. Factors that had univariable P value <0.2 and those considered to be a priori confounding variables (age, parity, duration of infertility, cause of infertility, number of oocytes retrieved, number of embryos generated and treatment type) were used to build the multivariable model in a stepwise regression modelling to adjust for confounding and assess the effect of follicular fluid microorganisms on the fertilization rate. Similar regression modelling was conducted for relationship between follicular fluid colonization and pregnancy using age, body mass index, parity, duration of infertility, cause of infertility, treatment protocol, number of oocytes retrieved, number of embryos generated and treatment type as the confounding variables. A P value <0.05 (95% confidence interval) was considered as statistically significant.

### Ethics statement

Ethical approval was obtained from the Institutional Review Board (IRB) committee of the National Hospital Abuja, Abuja, Nigeria before initiation of the study protocol (Approval No.: NHA/EC/070/2017). Written informed consent was obtained from all participants for their vaginal swabs and follicular fluids to be used in this study and gave permission for researchers to access medical records to obtain their reproductive history and IVF outcomes.

## Results

Of the 90 women that had IVF-ET treatments and were enrolled into the study, 96% (n = 86/90) completed the study. The participants had a mean age of 35 (± 3.5) years, mean BMI of 28 (± 4.9) kg/m^2^ while 47% (n = 42/86) were overweight and 81% (n = 73/86) were nulliparous. The median duration of infertility was 7 years (IQR; 1–20 years) and about 57% (n = 51/86) had secondary infertility and tubal factor accounted for 30% (n = 27/86) of the causes of infertility. Fifty-seven percent (n = 51/86) of the women had IVF while 39% (n = 35/86) had ICSI. Sixty-two percent of the women had down-regulation through GnRH agonist long protocol (n = 56/86) and about 1% of the women developed ovarian hyperstimulation syndrome (n = 1/86). The mean number of oocytes retrieved was 9 (± 6.0), the mean number of embryos generated was 6 (± 4.0) while the mean number of embryos transferred was 3.0 (± 1.0).

**Fig 1:**
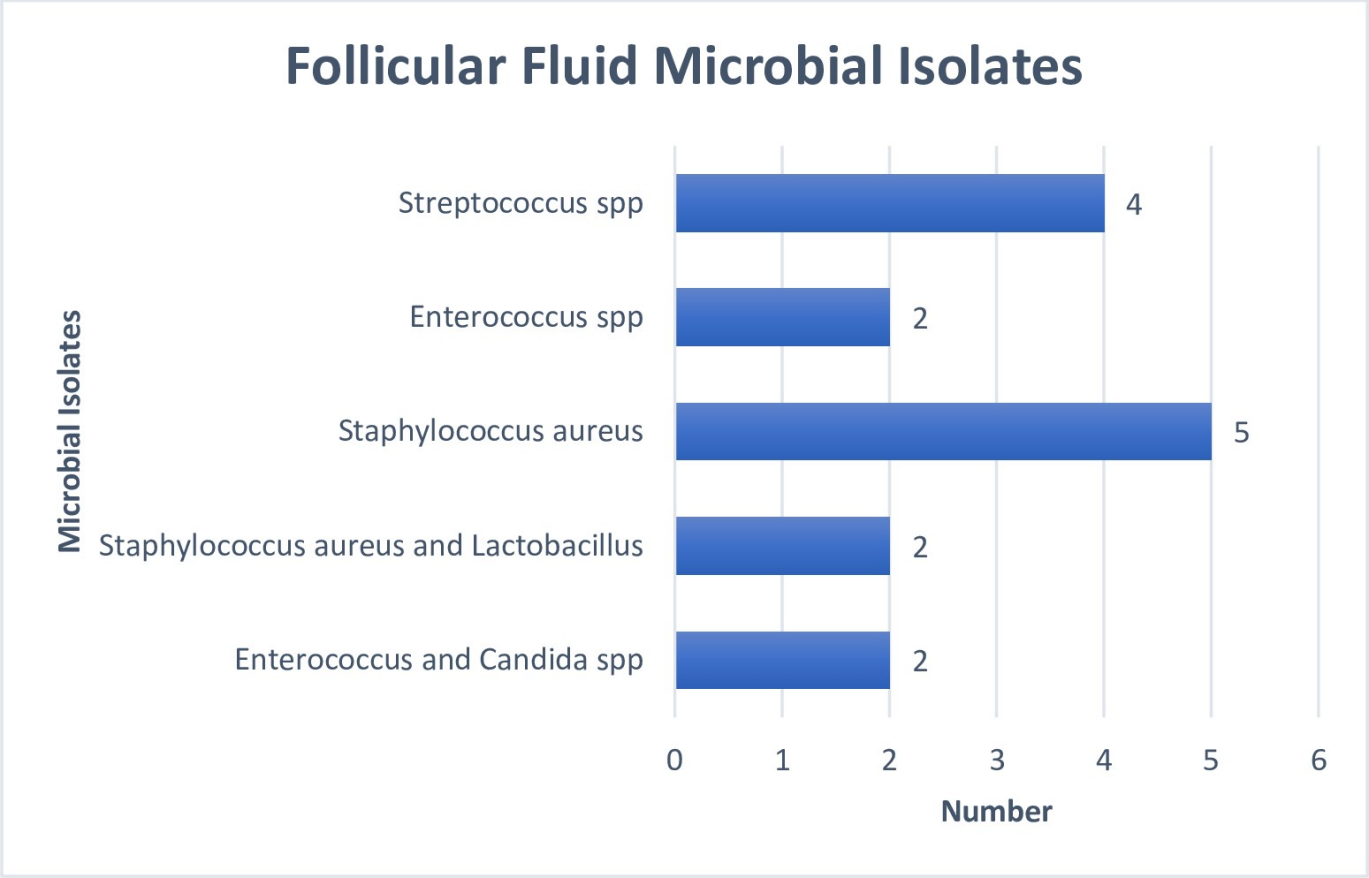
Frequency distribution of the follicular fluid microbial isolates.

Microorganisms were isolated in 53% (n = 48/86) of the HVS cultured and the organisms include; Streptococcus spp., *Staphylococcus aureus*, Enterococcus spp., Lactobacillus spp., Acinetobacter spp., *Klebsiella pneumonia*, *Escherichia coli* and *Candida albicans*. Microbial growths were also observed in 17% (n = 15/86) of the follicular fluids culture while contamination occurred in another seventeen percent (n = 15/86) of the cultured follicular fluids and the contaminants isolated were mainly diphtheroids (n = 10/15), *Staphylococcus aureus* (n = 3/15), and *Staphylococcus epidermidis* (n = 2/15). Microorganisms isolated from only follicular fluids were; Streptococcus spp. (n = 4/15), *Staphylococcus aureus* (n = 7/15), Enterococcus spp. (n = 4/15), Lactobacillus species (n = 2/15) and *Candida albicans* (n = 2/15) as illustrated in Fig 1.

Table 1 shows that there was no significant difference between the two groups of follicular fluid colonization (positive and negative) in terms of age, body mass index, parity, duration of infertility, type of infertility, cause of infertility, treatment protocol, type of treatment, fertilization rate, number of retrieved oocytes biochemical pregnancy rate and clinical pregnancy rate. There was however, a significant difference between the two groups in terms of number of generated embryos (P<0.001).

**Table 1:** Association between follicular fluid colonization and different variables.

Covariates (n = 86)	Follicular Fluid Colonization		P value
	Positive	Negative	
**Age group (years)**			
Under 35	3 (20)	25 (35)	0.25
35 and above	12 (80)	46 (65)	
**Body Mass Index**			
Normal weight	4 (27)	14 (20)	0.29
Overweight	9 (60)	33 (46)	
Obese	2 (13)	24 (34)	
**Parity**			
Nulliparous	12 (80)	60 (85)	0.50
Primiparous	3 (20)	8 (11)	
Multiparous	0 (0)	3 (4)	
**Duration of Infertility**			
Under 5 years	3 (20)	28 (40)	0.08
5–10 years	10 (67)	25 (35)	
10 years and above	2 (13)	18 (25)	
**Type of Infertility**			
Primary	7 (47)	32 (45)	0.91
Secondary	8 (53)	39 (55)	
**Cause of Infertility**			
Cervico-uterine	1 (7)	10 (14)	0.47
Male	0(0)	11(15)	
Tubal	5 (33)	22 (31)	
Ovarian	5 (33)	16 (23)	
Unexplained	2 (13)	4 (6)	
Others	2 (13)	8 (11)	
**Protocol**			
Long	9 (60)	45 (63)	0.81
Short	6 (40)	26 (37)	
**Treatment Type**			
IVF	10 (67)	41 (58)	0.52
ICSI	5 (33)	30 (42)	
**No. of Oocytes Retrieved**			
1–3	5 (33)	7 (10)	0.06
4–10	6 (40)	36 (51)	
>10	4 (27)	28 (39)	
**No. of Embryos Generated**			
1–3	9 (60)	14 (20)	0.00
4–10	6 (40)	47 (66)	
>10	0 (0)	10 (14)	
**Fertilization Rate**			
≤50%	3 (20)	9 (13)	0.46
>50%	12 (80)	62 (87)	
**Biochemical Pregnancy Rate (Serum Beta hCG level)**			
<200ng/ml	10 (67)	56 (79)	0.31
≥200ng/ml	5 (33)	15 (21)	
**Clinical Pregnancy Rate (Gestational sacs on ultrasound scan)**			
Absent	11 (73)	56 (79)	0.75
Present	4 (27)	15 (21)	

The average fertilization rate among women with positive follicular fluid colonization was found to be 81% while that among women with negative follicular fluid colonization was 64%. High fertilization rate (>50%) was seen in 80% of women with positive colonization and 87% of women with negative colonization. The biochemical pregnancy rate among women with positive follicular fluid colonization was 33% while that among women with negative colonization was 21%. The clinical pregnancy rate among women with positive colonization was 27% while that among women with negative colonization was 21%.

There was no significant association between follicular fluid colonization and fertilization rate after adjusting for the effect of age, parity, duration of infertility, cause of infertility, number of oocytes retrieved, number of embryos generated and treatment type (adjusted odds ratio [AdjOR] 0.55, 95% confidence interval [CI] 0.04–7.34; P = 0.10) (Table 2). Similarly, there was no significant association between follicular fluid colonization and pregnancy rate after adjusting for the effect of age, body mass index, parity, duration of infertility, cause of infertility, treatment protocol, number of oocytes retrieved, number of embryos generated and treatment type (AdjOR 4.02, 95% CI 0.56–28.92; P = 0.19) (Table 3).

**Table 2:** Logistic regression analysis showing the odds of follicular fluid colonization affecting fertilization rate and associated factors among the study participants.

Covariates	Crude (Unadjusted)			Adjusted*		
	Odds Ratio	95% CI	P value	Odds Ratio	95% CI	P value
**Follicular Fluid Colonization**						
Negative	1.00		0.48	1.00		0.10
Positive	0.58	0.13–2.46		0.55	0.04–7.34	
**Age group (years)**						
Under 35	1.00		0.57	1.00		0.21
35 and above	0.39	0.08–1.91		1.21	0.10–15.12	
**Parity**						
Nulliparous	1.00		0.37	1.00		0.55
Primiparous	1.76	0.20–15.60		13.94	0.47–409.81	
Multiparous	0.28	0.02–3.49		0.006	0.00–0.94	
**Duration of Infertility**						
Under 5 years	1.00		0.57	1.00		0.73
5–10 years	1.57	0.12–2.58		0.54	0.05–6.12	
10 years and above	0.61	0.11–3.37		0.39	0.02–6.19	
**Cause of Infertility**						
Cervico-uterine	1.00		0.28	1.00		0.48
Male	1.54	0.20–11.87		0.22	0.01–4.64	
Tubal	3.38	0.54–21.00		3.01	0.15–59.19	
Ovarian	9.03	0.77–106.08		4.97	0.16–154.39	
Unexplained	2.42	0.17–33.11		3.43	0.03–389.26	
Others	1.69	0.21–13.51		0.85	0.38–19.18	
**No. of Oocytes Retrieved**						
1–3	1.00		0.22	1.00		0.47
4–10	1.00			1.00		
>10	1.63	0.44–6.06		0.36	0.04–2.97	
**No. of Embryos Generated**						
1–3	1.00		0.84	1.00		0.42
4–10	1.75	0.45–6.71		26.12	2.25–303.67	
>10	1.00			1.00		
**Treatment Type**						
ICSI	1.00		0.54	1.00	0.00–0.77	0.05
IVF	0.25	0.05–1.24		0.05		

*Adjusted for age, parity, duration of infertility, cause of infertility, number of oocytes retrieved, number of embryos generated and type of treatment.

**Table 3:** Logistic regression analysis showing the odds of follicular fluid colonization affecting pregnancy rate and associated factors among the study participants.

Covariates	Crude (Unadjusted)			Adjusted*		
	Odds Ratio	95% CI	P value	Odds Ratio	95% CI	P value
**Follicular Fluid Colonization**						
Negative	1.00		0.32	1.00		0.19
Positive	1.87	0.55–6.29		4.02	0.56–28.92	
**Age group (years)**						
Under 34	1.00		0.26	1.00		0.35
35 and above	0.60	0.21–1.73		0.52	0.08–3.29	
**Body Mass Index**						
Normal weight	1.00		0.36	1.00		0.87
Overweight	0.81	0.23–2.87		2.50	0.29–21.89	
Obese	0.68	0.16–2.85		2.72	0.23–32.10	
**Parity**						
Nulliparous	1.00		0.24	1.00		0.34
Primiparous	0.27	0.03–2.30		0.29	0.02–5.38	
Multiparous	1.73	0.14–20.60		2.97	0.03–299.96	
**Duration of Infertility**						
Under 5 years	1.00		0.22	1.00		0.24
5–10 years	0.44	0.13–1.43		0.18	0.10–1.65	
10 years and above	0.36	0.09–1.55		0.06	0.00–0.82	
**Cause of Infertility**						
Cervico-uterine	1.00		0.39	1.00		0.75
Male	0.63	0.08–4.79		0.71	0.04–12.62	
Tubal	0.43	0.08–2.41		0.17	0.01–2.12	
Ovarian	1.22	0.24–6.23		0.62	0.05–8.22	
Unexplained	1.16	0.13–10.38		0.51	0.02–15.07	
Others	0.62	0.08–4.85		2.07	0.14–31.40	
**Treatment Protocol**						
Long	1.00		0.28	1.00		0.05
Short	0.33	0.10–1.10		0.28	0.04–1.81	
**No. of Oocytes Retrieved**						
1–3	0.57	0.10–3.33	0.22	0.11	0.00–2.68	0.53
4–10	1.00			1.00		
>10	1.47	0.50–4.31		1.29	0.27–6.30	
**No. of Embryos Generated**						
1–3	1.00		0.19	1.00		0.32
4–10	1.25	0.34–4.59		0.42	0.03–5.25	
>10	3.61	0.62–20.99		3.00	0.07–125.82	
**Treatment Type**						
ICSI	1.00		0.38	1.00		0.03
IVF	3.46	1.04–11.53		5.66	0.93–34.27	

*Adjusted for age, BMI, parity, duration of infertility, cause of infertility, treatment protocol, number of oocytes retrieved, number of embryos generated and type of treatment

## Discussion

This study demonstrates that human follicular fluid can be colonized by microorganisms and hence, not always sterile. Bacteria and fungi were isolated from the follicular fluid collected during transvaginal oocyte retrieval in women undergoing IVF-ET treatment cycles. There was no evidence of association between the presence of these colonizing microorganisms (Streptococcus spp., *Staphylococcus aureus*, Enterococcus spp., Lactobacillus species and *Candida albicans)* and the IVF outcomes; fertilization and pregnancy rates. However, significant association was found between the presence of microorganisms within the FF and the number of generated embryos.

A similar study reporting the presence of microorganisms within human follicular fluid showed the percentage of women with colonized follicular fluid to be 34.4% [21]. which is much higher than our finding of 17%. Previous studies have also reported follicular fluid colonization of 24% to 37% based on the aetiology of infertility [8] as well as 40% and 32% from left and right ovaries respectively [22]. The discrepancies between present finding and previously published data may be attributed but not limited to the differences in methodology, sample size and within the demographic and clinical features of the participants.

The organisms considered as follicular fluid colonizers were not simultaneously present within the vagina, suggesting that they were not likely to be introduced as part of the IVF procedure, but may have colonized the FF independently. Some of the microorganisms isolated were similar to those isolated by Pelzer et al (Streptococcus spp., Staphylococcus spp., and Lactobacillus) [8]. Considerably, these colonizers may represent an extension of the normal regional flora of the lower genital tract and transient flora of the uterine cavity or rather, a continuum from the lower to the upper genital tract. Notwithstanding, the organisms detected in both the FF and vaginal secretions of the same participant may actually be contaminating organisms transferred into the FF during oocyte retrieval [7].

This study has demonstrated that there was no significant difference in the type of infertility between the positive and negative FF colonization groups. This is however, contrary to findings from a cross-sectional study in Iraq, where secondary infertility was significantly associated with FF colonization [21]. No difference was found between the two groups in the cause and duration of infertility in this study which is similar to findings by Hamad et al. [21].

Although, the findings of this study showed that there was no evidence of association between the presence of follicular fluid microorganisms and the number of retrieved oocytes, a significant association with the number of generated embryos was found at univariate level of analysis. This finding may be related to the microorganisms’ metabolic activity and their ability to produce reactive oxygen radicals associated with oxidative stress and poor oocytes quality [23, 24]. While Noor et al [25] and Kim et al [26] reported similar findings, Cottell et al demonstrated a significant decrease in the number of retrieved oocytes from women when microorganisms were isolated from their follicular fluids [22].

The multivariable logistic regression analysis illustrates that there was no evidence of significant difference in the fertilization and pregnancy rates between the positive and negative FF colonization groups, even after adjusting for the effect of other variables. This suggests that FF colonization does not affect fertilization and pregnancy rates following IVF/ICSI treatments which is found to be in agreement with the findings of other studies [8, 22]. On the contrary, larger studies have reported a decrease in fertilization rates, embryo transfer rates, clinical pregnancy rates, and an increase in embryo discard rates with follicular fluid colonization [2, 17]. Interestingly, *Lactobacillus specie* as a contaminant was associated with positive pregnancy outcome in a study by Pelzer et al. [8].

The primary strengths of this study were the availability of a reputable IVF unit where facility-related and procedure-related adverse effects on IVF/ICSI outcomes are unlikely. The study population and inclusion criteria were clearly defined and confounding variables controlled for by multivariable analysis. Furthermore, there was good representation, therefore findings can be generalised to the target population. The study was however, limited by the paucity of data on the subject matter especially in our environment which hindered adequate comparison with the results obtained. Challenges with molecular identification techniques might have limited the identification of some species of microorganisms.

The results of this analysis are a valuable addition to the huge knowledge gap on the effect of FF microorganisms on IVF/ICSI outcomes in our environment. These will be of interest to Gynaecologists and other experts involved with fertility treatments especially during counselling of women prior to IVF/ICSI. The need for large, multi-centre studies cannot be over-emphasized as this study has provided an important opportunity to advance the understanding of microorganisms in fertility treatments.

Further studies may consider using molecular techniques for better identification of some microorganisms. In addition, differentiation of follicular fluids from the left and right ovary, and stratification of the analysis based on each microorganism may give better insight to the associations studied. Strong consideration should be given to women going for IVF, especially those with previous failed IVF treatments and those with unexplained infertility as they may benefit from microbial screening of vaginal swabs prior to entry into an IVF/ICSI treatment cycle to detect abnormal vaginal flora, and by culturing the follicular fluid collected at the time of trans-vaginal oocyte retrieval for the presence of microorganisms.

## Conclusions

The presence of microorganisms in the follicular fluid collected during trans-vaginal oocyte retrieval of women undergoing IVF/ICSI treatment has been demonstrated, both as colonization and contamination. However, this was not found to adversely affect fertilization and pregnancy rates following IVF/ICSI treatment cycles.

## Supporting information

S1 Text Questionnaire. (DOCX)

S2 Text Sample size calculation. (DOCX)

S1 Data Minimal anonymized data set. (XLSX)

S1 Raw images Microscopy images of bacteria and fungi isolated. (ZIP)
