# Supplementary material for: The presence of microorganisms in follicular fluid and its effect on the outcome of in vitro fertilization-embryo transfer (IVF-ET) treatment cycles
Source: PLoS One. 2021 Feb 8;16(2):e0246644. doi: 10.1371/journal.pone.0246644 (PMC7870083; doi:10.1371/journal.pone.0246644)
Supplement: S1 Text — (DOCX) [file pone.0246644.s001.docx]

# QUESTIONNAIRE

**Study Title:** The presence of microorganisms in follicular fluid and its effect on the outcome of in vitro fertilization-embryo transfer (IVF-ET) treatment cycles

Serial number: -------------------------------------

Phone number: ------------------------------------

***General characteristics of the participant:***

- Hospital number: -----------------------------
- Age (years): -----------------------------------
- Educational Status: -------------------------
- Ethnicity: ---------------------------------------
- Weight: -----------------------------------------
- Height: -----------------------------------------
- BMI: --------------------------------------------

***Reproductive Characteristics of the participant:***

- Parity: -------------------------------------------
- Duration of infertility (years): --------------
- Type of infertility (tick as appropriate):
- Primary
- Secondary
- Cause of infertility (Indication for IVF):
- Tubal factor
- Ovarian Factor
- Unexplained infertility
- Male Factor
- Other factors (Please specify)

***Characteristics of the IVF cycle***:

- Type of protocol:
  - Long
  - Short
- Treatment type:
  - IVF
  - ICSI
- Number of oocytes retrieved: ----------------------
- Number of embryos generated: -------------------
- Fertilization Rate: -------------------------------------
- Number of embryos transferred: ------------------
- Reason(s) for Cycle cancellation;
- Poor Response: -------------------------------
- No oocyte retrieved: --------------------------
- Fertilization failure: ----------------------------
- Other (please specify): -----------------------
- Complication(s) encountered during treatment;
- Intravenous sedation: --------------------------
- Pelvic infection: ----------------------------------
- OHSS: ---------------------------------------------
- Others (please specify): -----------------------

***Microbiological Results:***

- High vaginal swab: ---------------------------------------
- Follicular fluid: ---------------------------------------------

***Pregnancy outcome:***

- Pregnancy test (Plasma hCG level ≥ 200iu/L on day 14 post embryo transfer)
  - Positive: --------------------------------------
  - Negative: ------------------------------------
- Ultrasound scan evidence of gestational sac(s) two weeks after pregnancy test
  - Number of gestational sacs seen: -------------------------
