# Supplementary material for: The presence of microorganisms in follicular fluid and its effect on the outcome of in vitro fertilization-embryo transfer (IVF-ET) treatment cycles
Source: PLoS One. 2021 Feb 8;16(2):e0246644. doi: 10.1371/journal.pone.0246644 (PMC7870083; doi:10.1371/journal.pone.0246644)
Supplement: S2 Text — (DOCX) [file pone.0246644.s002.docx]

## Sample Size Calculation

The minimum sample size (n) was determined by applying the formula for comparison of two proportions[1, 2]. This sample size takes into account the required significance level and power of the test (95% confidence level and 80% power):

n= (Z_α_ + Z_β_)^2^{P_1(1OO-_P_1) +_ P_2 (100-_P_2)_}

(P_1-_P_2_)^2^

n =minimum sample size

P_1_ = The clinical pregnancy rate per transfer in women with non-colonized follicular fluid (28% from a previous study)[3]

P_2_ = The clinical pregnancy rate per transfer in women with colonized follicular fluid (58% from a previous study)[3]

Z_α_ = Standard normal deviate corresponding to the level of significance

Z_β_ = Standard normal deviate corresponding with 1 minus power

At 95% confidence level, with 90% power, Z_α_ =1.96 and Z_β_ =1.28

n= (1.28 + 1.96)^2^ {28 (100-28) + 58 (100-58)}

(28 – 58)^2^

n = 39

Assuming equal number of patients in both arms; n = 78

10% Attrition rate = 8

n = 78 + 8 = 86

The sample size was approximated to 90 patients.

10% has been chosen as attrition to account for loss to follow-up, sampling/labelling and specimen processing errors.

References:

[1] Kirkwood BR, Sterne JA. Calculation of required sample size. In: *Essential Medical Statistics*. Massachusetts: Blackwell Publishing Company, 2016, pp. 413–428.

[2] Whitley E, Ball J. Statistics review 4: sample size calculations. *Crit Care* 2002; 6: 335–41.

[3] Pelzer ES, Allan JA, Waterhouse MA, et al. Microorganisms within Human Follicular Fluid: Effects on IVF. *PLoS One*; 8. Epub ahead of print 2013. DOI: 10.1371/journal.pone.0059062.
