# Supplementary figures and images for: The presence of microorganisms in follicular fluid and its effect on the outcome of in vitro fertilization-embryo transfer (IVF-ET) treatment cycles
Source: PLoS One. 2021 Feb 8;16(2):e0246644. doi: 10.1371/journal.pone.0246644 (PMC7870083; doi:10.1371/journal.pone.0246644)

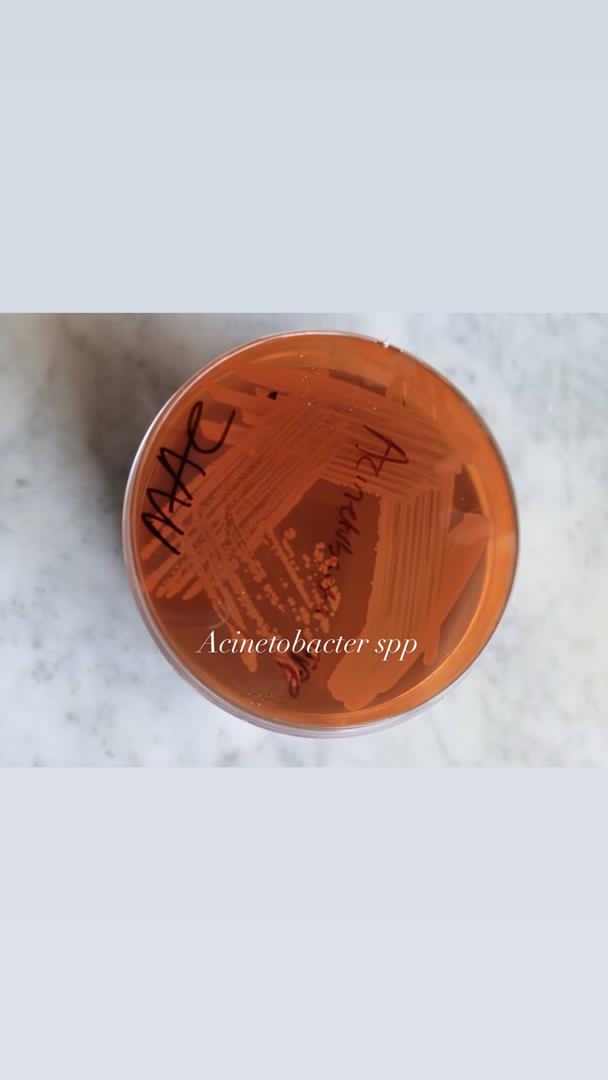

Supplement: S1 Raw images — (ZIP) [file pone.0246644.s004.zip › Acinetobacter spp (Isolated from HVS)-min.jpg]

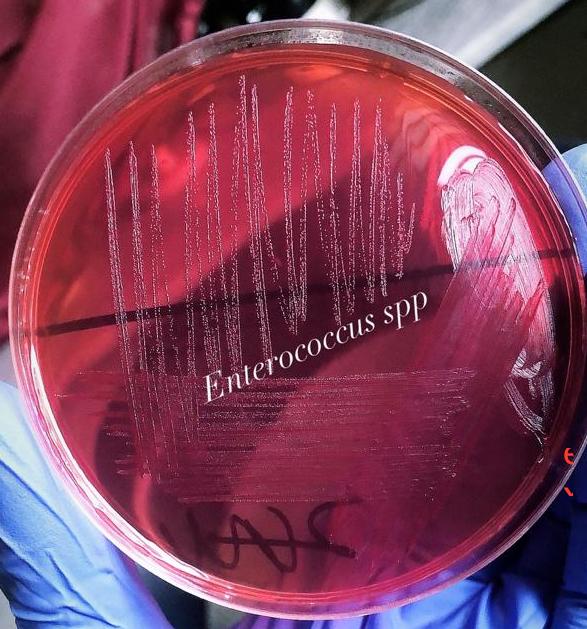

Supplement: S1 Raw images — (ZIP) [file pone.0246644.s004.zip › Enterococcus spp (Isolated from both HVS & FF)-min.jpg]

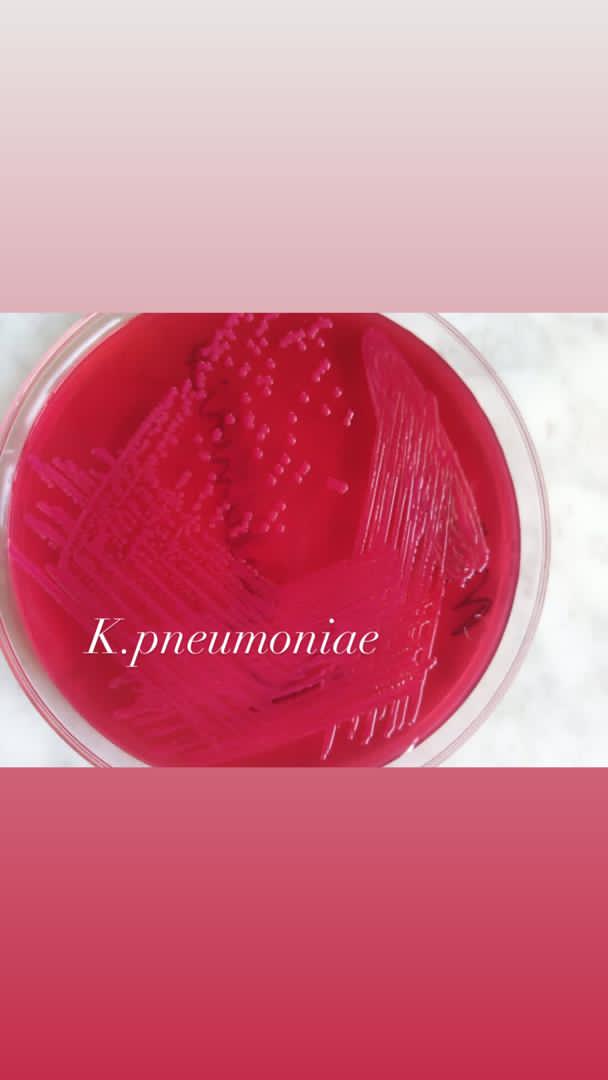

Supplement: S1 Raw images — (ZIP) [file pone.0246644.s004.zip › Klebsiella pneumoniae 2 (Isolated from HVS)-min.jpg]

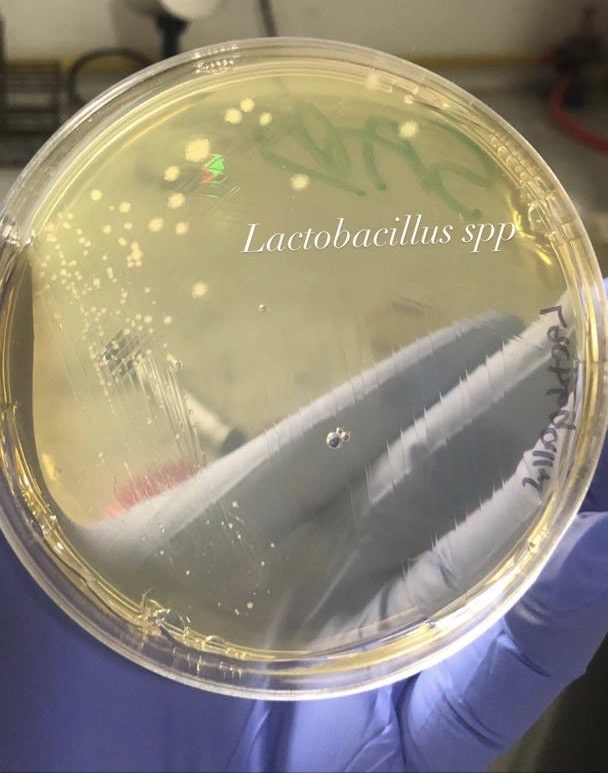

Supplement: S1 Raw images — (ZIP) [file pone.0246644.s004.zip › Lactobacillus spp.JPG (Isolated from both HVS & FF)-min.JPG]

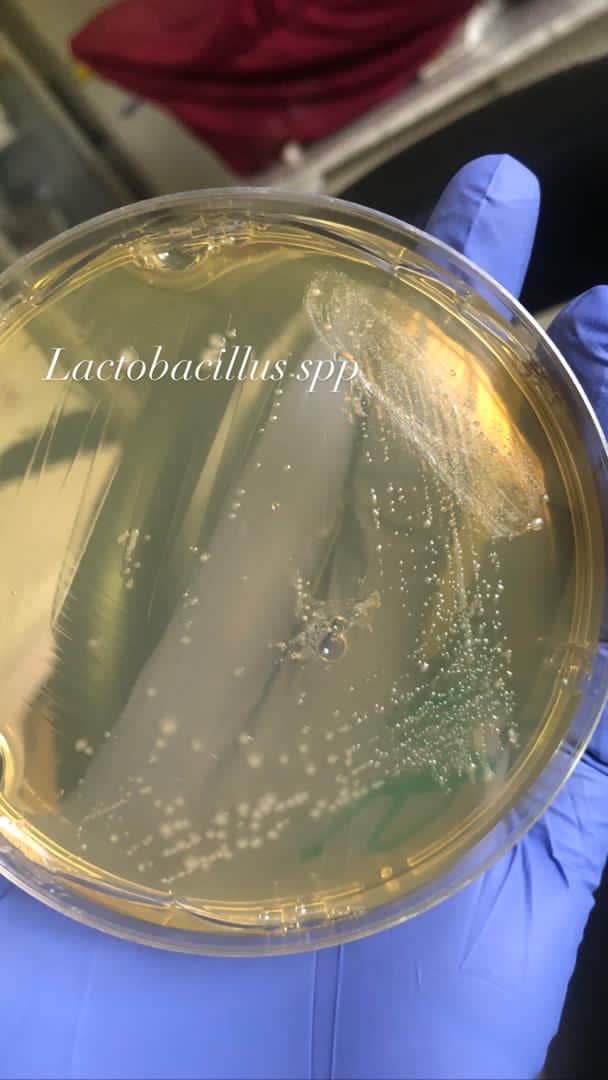

Supplement: S1 Raw images — (ZIP) [file pone.0246644.s004.zip › Lactobacillus spp2.JPG (Isolated from both HVS & FF)-min.JPG]

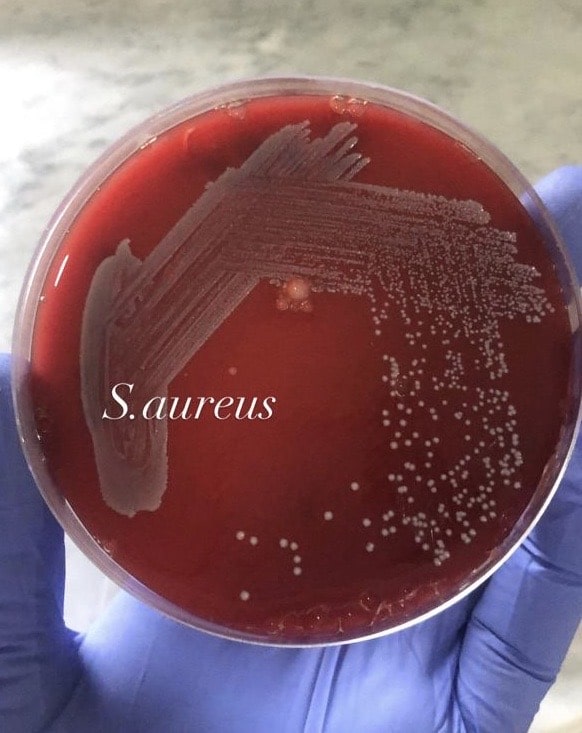

Supplement: S1 Raw images — (ZIP) [file pone.0246644.s004.zip › Staphylococcus aureus (Isolated from both HVS & FF)-min.JPG]

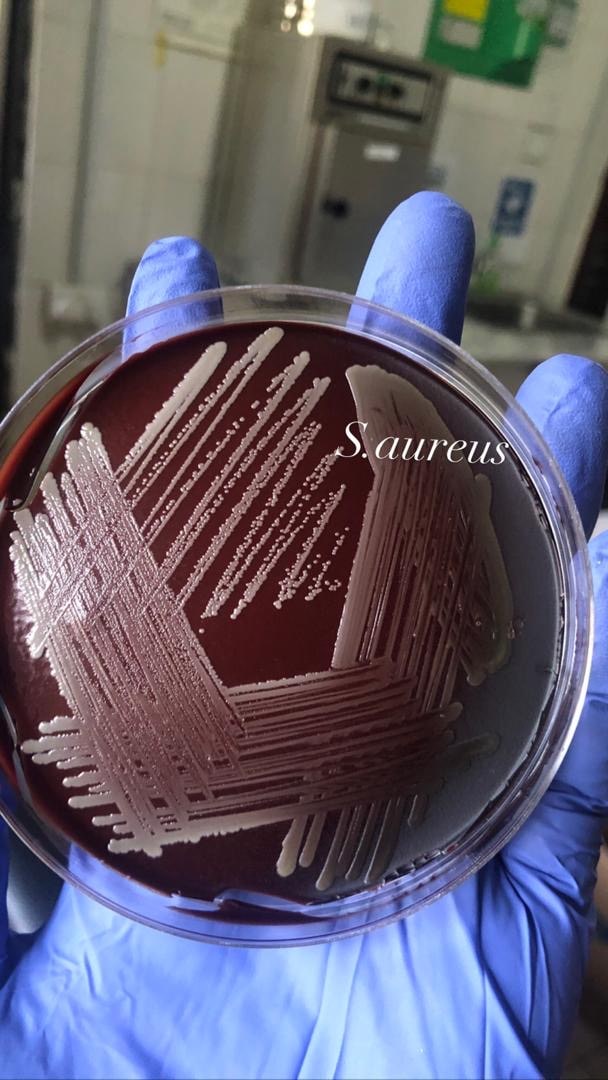

Supplement: S1 Raw images — (ZIP) [file pone.0246644.s004.zip › Staphylococcus aureus 2 (Isolated from both HVS & FF)-min.JPG]

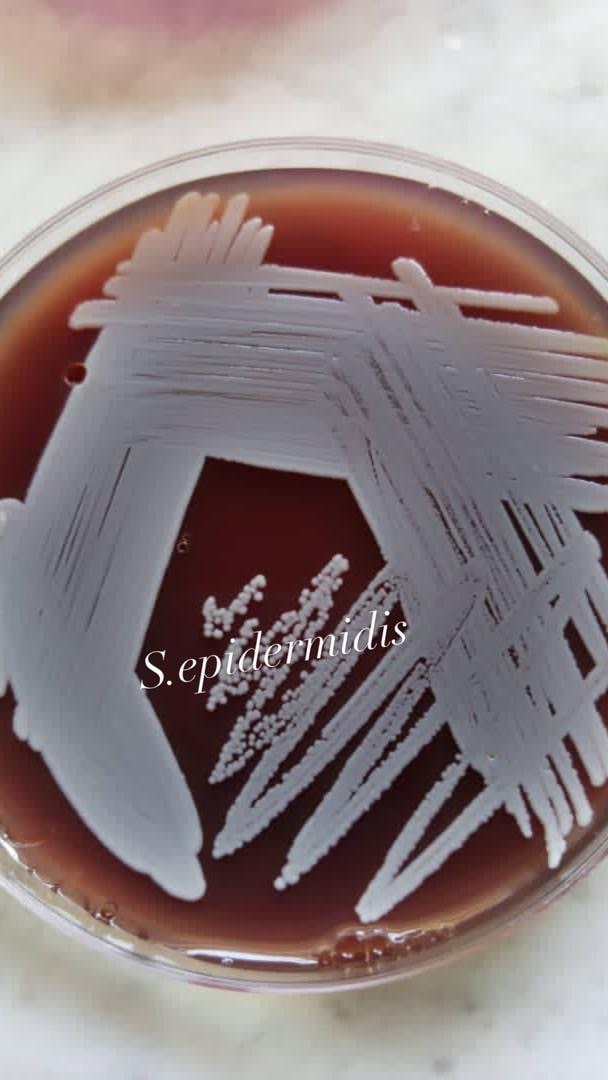

Supplement: S1 Raw images — (ZIP) [file pone.0246644.s004.zip › Staphylococcus epidermidis (Isolated as a contaminant)-min.JPG]

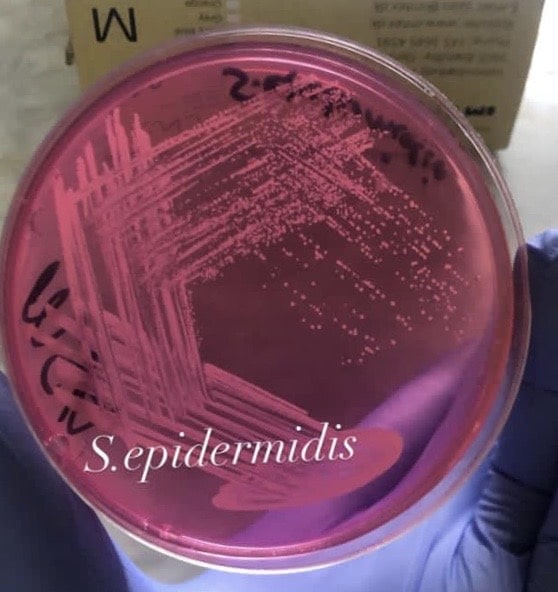

Supplement: S1 Raw images — (ZIP) [file pone.0246644.s004.zip › Staphylococcus epidermidis 2 (Isolated as a contaminant)-min.JPG]
